# Integrated poultry production as a reservoir of tet(X4) and mcr-1.1 encoding Escherichia coli in Pakistan

**DOI:** 10.1099/mgen.0.001800

**Published:** 2026-08-03

**Authors:** Muhammad Ahmed Mushtaq, Peter Schierack, Muhammad Yousaf, Fariha Fatima, Tayyaba Qamar, Mashkoor Mohsin, Muhammad Moman Khan

**Affiliations:** 1 Department of Multiparametric diagnostics, Brandenburg University of Technology, Cottbus-Senftenberg, Germany; 2 Institute of Animal and Dairy Sciences, University of Agriculture Faisalabad, Faisalabad, Pakistan; 3 Institute of Microbiology, University of Agriculture Faisalabad, Faisalabad, Pakistan

**Keywords:** antimicrobial resistance, *Escherichia coli*, farm-to-fork, *mcr-1.1*, poultry, *tet(X4)*

## Abstract

Plasmid-mediated colistin and tigecycline resistance threatens last-resort therapeutic options. We investigated the farm-to-fork dissemination of *Escherichia coli* encoding *mcr-1.1* and *tet*(X4) in a vertically integrated broiler production system. A total of 200 samples were collected in 2024, representing four sequential production stages: broiler breeders, day-old chicks, day-30 broilers and retail meat. All positive [*mcr-1.1* and/or *tet(X4*)] isolates underwent Illumina short-read sequencing, with long-read sequencing of three representatives. The study was enriched and analysed with *mcr-1.1* and *tet*(X4) encoding 213 publicly available *E. coli* genomes from Pakistan. Of 18 isolates, *tet*(X4) was detected in 11 and *mcr-1.1* in 7 isolates with a single breeder isolate (ST-398) co-harbouring both on separate plasmids. *tet*(X4) predominated breeders and day-30 broilers, whereas *mcr-1.1* was distributed across all four stages. ST-1011 exhibited a pattern of clonal farm-to-fork transmission of *tet*(X4) via IncFIB(AP001918)-IncFII providing molecular evidence suggestive of clonal transmission. Analysis of 231 genomes across 87 sequence types (STs) revealed contrasting evolutionary trajectories: *mcr-1.1* with post-mobilization stabilization marked by complete absence of ISApl1 and highly conserved IncI2 plasmid (93%), whereas *tet*(X4) retained active transposition within IS26-bounded elements. *tet*(X4) carriage was also associated with elevated resistance gene burden, driven by the IncF megaplasmids. All 18 isolates exhibited adhesion to chicken intestinal epithelial cells (CHIC-8E11), with high adhesion capacity among *tet*(X4)-positive isolates. This study demonstrates that *mcr-1.1* and *tet*(X4) disseminate through poultry production via distinct mechanisms. These findings highlight the need for integrated One Health surveillance of antimicrobial resistance dissemination.

**Impact Statement**

The global dissemination of plasmid-mediated resistance to colistin and tigecycline, two last-line antibiotics, pose a growing One Health threat. Despite South Asia being one of the world’s largest poultry-producing regions, genomic evidence describing the farm-to-fork dissemination and evolution of these resistance determinants remains limited. This study fills a critical regional knowledge gap by providing a comprehensive genomic investigation of *Escherichia coli* carrying *mcr-1.1* and *tet*(X4) across a vertically integrated broiler production system in Pakistan, complemented by nationwide comparative genomic analysis. Our findings demonstrate distinct evolutionary strategies for these two clinically important resistance determinants. While *mcr-1.1* appears to have undergone post-mobilization stabilization through highly conserved IncI2 plasmids, *tet*(X4) remains associated with dynamic IS26-mediated mobile elements carried on multiple drug-resistant IncF megaplasmids. Genomic evidence consistent with clonal dissemination from broiler breeders to retail meat highlights the capacity of these clinically important resistance determinants to reach consumers through the food chain. The detection of related resistance lineages in wild birds further supports the role of environment in the wider spread of these mobile resistance genes.

This study provide evidence to support national poultry antimicrobial stewardship policies and integrated One Health surveillance for tracking the spread of critically important antimicrobial resistance in Pakistan and beyond.

## Data Summary

The data presented in the study are deposited in the online repository NCBI BioProject database under the accession no. PRJNA1253608. Biosample accession numbers for each isolate are listed in the Table S1. The four assembled plasmid sequences are also submitted to the NCBI database with the GenBank submission: SUB16241252.

## Introduction

Antimicrobial resistance (AMR) has now become an established, most pressing global health problem and has been recognized as one of the top ten global health threats by major organizations such as WHO, FAO and other regional and international health institutes [1, 2]. It poses grave ramifications for both human and animal health along with considerable social repercussions [3]. The economic burden of AMR is significant, with annual financial loss estimated at 1.5 billion EUR in the European Union and 53 billion to 3 trillion USD globally, related to nosocomial infections [4]. Additionally, intensifying the problem, forecasts indicate that by 2050, AMR could cause up to 10 million deaths annually and impose a global economic burden of nearly 100 trillion USD [5]. Within this broader impact, the cumulative loss of economic production for the Organization for Economic Cooperation and Development countries alone is projected to reach 20–35 trillion USD, posing financial and economic strain on high-income countries [4, 6]. This ‘silent pandemic’ requires immediate and thorough surveillance and monitoring strategies to enable prompt reporting for informed and effective policymaking [7, 8].

AMR represents a critical challenge at the convergence of human, animal and environmental health, a concept underscored by the One Health approach. This approach acknowledges the required interrelation of these sectors and emphasizes the importance of integrated monitoring and surveillance [9]. The primary cause is the unsupervised and irregular use of antimicrobials for prophylactic, growth promotion or treatment purposes [10]. In low- and middle-income countries, the challenges are exacerbated by the widespread use of antibiotics in livestock, especially in poultry farming, particularly where antibiotic stewardship practices are often unregulated [11]. Moreover, antimicrobial usage in poultry production when compared to the other sectors is notably high and is projected to continue on an upward trajectory until 2040 [12]. For example, studies indicate that incorrect application and usage of antibiotics in agriculture, specifically in livestock, plays a significant role in the selection and spread of AMR-associated pathogenic strains, e.g. *Escherichia coli,* within the food chain [13]. Similarly, water treatment facilities that are contaminated with antibiotic residues from clinical wastes, runoff from livestock farms and anthropogenic activities can disseminate AMR, creating reservoirs of mobile genetic elements (MGEs) and antibiotic resistance genes (ARGs) impacting various ecological niches [14]. This has created a chain of AMR transmission from farm-to-fork that is expanding at an exponential rate, contaminating the entire food chain [15]. It is also causing the livestock sector to decline in productivity due to increased cases of hard-to-treat AMR infections, making AMR a significant challenge for both health care and food security [16]. The main stakeholder here is the poultry sector especially in Pakistan as it is a major protein source in the region and also happens to be harbouring various ESKAPEE pathogens [17, 18].

This concern is especially heightened regarding last-resort antibiotics, such as tetracyclines and colistin, which remain indispensable for managing infections caused by multiple drug-resistant (MDR) organisms [19]. Despite their restricted role as a last-resort therapy, colistin- and tetracycline-resistant bacteria continue to circulate among human- and animal-associated bacterial strains due to their plasmid-mediated mobility enabling efficient horizontal gene transfer [20]. As AMR continues to evolve, colistin resistance which started spreading via the plasmid-borne route (*mcr-1.1* and its related variants) has now been observed to be stabilized in the plasmids by lowering the fitness cost, augmenting the fear of stability and is expected to drastically increase the spread of colistin resistance in food chain [21, 22]. Similarly, tetracycline resistance has also increased because bacteria acquire various conjugative plasmids that confer heteroresistance, mostly together with fosfomycin (FOS) (*fosA4*, *fosA3* etc.), thereby aggravating horizontal gene transfer [23]. Now, several bacterial strains have been identified as co-harbouring colistin, tigecycline and FOS resistance genes [24–27].

This study focused on *E. coli* isolates obtained from poultry at various production stages beginning with broiler breeders and extending to meat ready for the consumer market. To create a comprehensive genomic profile, the scope of the study was broadened by incorporating all *E. coli* genomes documented in Pakistan harbouring *mcr-1.1* and *tet*(X4) genes. The data offer significant insights into the spread of AMR in the poultry industry, presenting a comprehensive overview of the human–environment–animal triad within the AMR surveillance framework.

## Methods

### Sample collection and bacterial isolation

A total of 200 samples were collected from a vertically integrated commercial broiler production system in Punjab, Pakistan, in 2024 representing four sequential production stages: broiler breeders, day-old chicks, day-30 broilers and retail meat (*n*=50 per stage). Cloacal swabs were collected from live birds at farm stages, whereas retail meat samples were obtained from commercial outlets within the same production network. All samples were enriched in brain heart infusion broth at 37 °C for 18–24 h then inoculated onto UTI CHROMagar plates supplemented with either colistin (2 µg ml^−1^) or tigecycline (4 µg ml^−1^) and incubated at 37 °C for 24 h. Presumptive colonies were selected based on chromogenic morphology. Species identification was confirmed by MALDI-TOF MS (Bruker Daltonics, Germany).

### PCR detection of *mcr-1.1* and *tet*(X4)

Initially to confirm the presence of *mcr-1.1* and *tet*(X4), DNA of *E. coli* isolates was subjected to conventional PCR to screen the presence of these genes using primers and conditions, as described previously [28, 29].

### Antimicrobial susceptibility testing

Antimicrobial susceptibility was determined by disc diffusion for all agents except colistin, for which broth microdilution was used to determine the minimum inhibitory concentration (MIC). The list of antimicrobials is given in the Table S1, available in the online Supplementary Material. Results were interpreted using the dosage and breakpoints as mentioned in the European Committee on Antimicrobial Susceptibility Testing manuals (v 15.0) [30]. ATCC25922 was used as a negative control.

### Biofilm formation

All the strains were tested for various biofilm-forming conditions on three different growth media, i.e. brain heart infusion broth, Luria–Bertani (LB) broth and minimal media, with 48 h incubation at 37 °C. The quantification was done using a modified version of our previously established protocol. Biofilms were formed on 96-well flat-bottom polypropylene plates (Greiner Bio-One GmbH, Frickenhausen, Germany). *E. coli* strain K-12 MG1655 F’tet Δ*traD* served as a positive control for biofilm formation. Following the incubation, non-adherent bacteria were carefully aspirated and the wells containing biofilms were washed with 200 µl of sterile 0.9% NaCl solution. Subsequently, an incubation in the dark for 10 min was conducted using isotonic saline comprising 5 µM SYTO 9 green, fluorescent nucleic acid stain (Thermo Fisher Scientific GmbH, Dreieich, Germany). The plates were washed again with isotonic saline and then analysed using the automated VideoScan technology platform [31]. Experiments were repeated independently for each strain in three well per strain format.

### Adhesion assay

Dulbecco’s modified Eagle medium HAM’S/F-12 (1 : 1) (Biochrom, Berlin, Germany) media enriched with 10% FBS and 1% l-glutamine (Biochrom, Berlin, Germany) was used to grow chicken intestinal epithelial cells, CHIC-8E11 (MicroMol GmbH, Karlsruhe, Germany), in 96-well plates and maintained them in a humidified atmosphere containing 5% CO_2_ at 37 °C. Adhesion assays were carried out using the established method using the Aklides system (Medipan GmbH, Potsdam, Germany) as previously described [32, 33]. Bacterial adhesion was classified as (1) low (1–2,000 bacteria mm^−2^); (2) medium (2,001–4,000 bacteria mm^−2^) and (3) highly adherent (more than 4,000 bacteria mm^−2^). Assays were done in triplicate wells and were repeated at least three times.

### Whole-genome sequencing

Genomic DNA was extracted from overnight LB cultures of all *mcr-1.1*- and/or *tet*(X4)-positive isolates using the QIAcube automated system (Qiagen, Germany). DNA concentration and purity were assessed using the Qubit fluorometer (Thermo Fisher Scientific, USA). Short-read sequencing was commercially performed by Genewiz GmbH (Germany) using the NGS DNA Library Prep Set and the Illumina NovaSeq platform with paired-end reads (2×150 bp; PE150), with an approximate sequencing depth of 100×. Additionally, three representative isolates SAMN48080404, SAMN48080405 and SAMN48080415, as *de novo* (Nanopore) long-read sequencing with raw-data requirement of 1 Gb sample^−1^, using the SQK-LSK110 ligation library-preparation kit by Oxford Nanopore Technologies (ONT) platform (Novogene GmbH, Germany). Sequencing details are in Table S2.

### Bioinformatics analysis

#### Read quality control, trimming and short-read assembly

First for short-read sequencing, quality was checked with FastQC v0.12.1, and quality trimming was done using Trimmomatic v0.40 [34] in paired-end mode with Illumina TruSeq3 adapter clipping, leading/trailing low-quality base removal, sliding-window quality filtering and a minimum retained read length of 36 bp. Reads retained after trimming were assembled *de novo* using SPAdes v3.15.5 [35] and constituted working assemblies for the full isolate set. Per-isolate read counts, yield, coverage and quality summaries are provided in Table S3.

### Long-read assembly, polishing and hybrid assembly

For long read, Nanopore raw reads were basecalled with Dorado v0.7.2 [36] using the most efficient basecalling model dna_r10.4.1_e8.2_400bps_sup@v5.0.0. Read statistics, including read N50, were computed directly from the raw FASTQ files with seqkit [37]. Initial long-read filtering was performed with Filtlong (--min_length 1000, --keep_percent 90). For SAMN48080404, read-quality inspection indicated poor quality across the first nine bases, so the leading 9 bp were removed with Cutadapt (-u 9) before Filtlong filtering (--min_length 1000, --keep_percent 50) and NanoFilt v2.8.0 [38] was applied with explicit thresholds (-q 10, -l 1000).

To estimate long-read depth, filtered ONT reads were mapped to the corresponding assembly with minimap2 (map-ont preset); alignments were sorted and indexed with SAMtools v1.2 [39]. Per-base depth was calculated using samtools depth -aa so that zero-coverage positions were included. ONT read statistics, read N50 and genome-wide depth are summarized in Table S4. Long-read assemblies were done by using Flye v2.9.6 [40] (--nano-raw, expected genome size 5 Mb [-g 5 m], --min-overlap 2000, 16 threads). Draft assemblies were polished for SAMN48080415, which required additional curation, Racon v1.5.0 [41] was applied for ~2–3 rounds and Medaka v2.0.1 [42] was used for ONT read-based consensus correction. For the three representative isolates, hybrid assemblies were subsequently produced with SPAdes v3.15.5 [35] from the filtered ONT reads together with the Illumina paired-end reads. The short-read assemblies and hybrid assemblies were used for full isolate set and for the main comparative genome analysis including downstream processing. Hybrid assemblies were mainly used for extracting plasmid architecture and reconstruction with AMR gene localization.

### Assembly quality control

Assembly quality was evaluated with Quast v5.3.0 [43] (total length, number of contigs, largest contig, contig N50, G+C content and gap statistics). Genome completeness was assessed with BUSCO v5.8.2 [44] in genome mode (--auto-lineage-prok); completeness was summarized against the bacteria_odb12 dataset, with Enterobacterales/Enterobacteriaceae summaries additionally consulted for assemblies. Completeness and contamination were also evaluated with CheckM2 v1.1.0 [45]. The NCBI foreign contamination screen [46] was run locally, and seqkit v2.10 [37] together with read-alignment evidence was used for additional sequence curation. Most final assemblies showed high completeness and low contamination after curation. SAMN48080409 remained fragmented, with reduced CheckM2/BUSCO quality and mixed coverage populations; retained cautiously in the core-genome phylogeny, where inference rests on conserved core-genome loci rather than total assembly content. SAMN48080415 was likewise treated cautiously at the whole-genome level, although its plasmid-level conclusions are supported by independent long-read boundary validation. Quality metrics are provided in Table S5.

### Genome annotation

High-quality curated assemblies were annotated with Bakta v1.9.4 [47] to generate standardized genome features. Antimicrobial-resistance genes were detected using the Resistance Gene Identifier v6.0.5 with the Comprehensive Antibiotic Resistance Database v4.0.1 [48] and ResFinder [49]; ARG counts were defined as the number of unique detected ARG features per genome after curation. Virulence-associated genes (VAGs) were detected with ABRicate using the virulence factor database (VFDB) (2,597 nucleotide sequences; 19 June 2025 version), and VAG counts were defined as unique VFDB gene names per strain after deduplication, and the same method was utilized for ARGs. Full ARGs and VAGs presence-absence matrices for 231 genomes are available in Tables S10 and 11, respectively. Source-group comparisons of ARG and VAGs counts were performed. MGEs and plasmid-related features were investigated using MGEfinder [50], PlasmidFinder [51], geNomad [52], ISEScan [53], ISfinder [54] and MOB-suite v3.1.9 [55] which assigned plasmid identity, replicon type, relaxase/mating pair formation type, mobility class, nearest neighbour and cluster.

### Plasmid reconstruction

Plasmid-associated contigs were identified by MOB-suite and assembly-context analysis, followed by full plasmid reconstruction. For SAMN48080415, unresolved plasmid sequences spanning multiple contigs were scaffolded with reference-guided RagTag v2.1.0 [56]. pEC9116A [carrying *tet*(X4)] was scaffolded against the reference plasmid pEC8331-tetX, and pEC9116B (carrying *mcr-1.1*) against pZJ3920-3 from *E. coli* ZJ3920 (CP020548.1). RagTag was used only for this plasmid reconstruction and not as a general genome-assembly method. Fully annotated plasmid tables for the hybrid assemblies were generated using custom Python scripts, and regions shared across plasmids and the broader genomic context were visualized using the blast Ring Image Generator (BRIG) [57].

Circularization of the reconstructed plasmids was assessed by ONT long-read boundary mapping. For each plasmid, a boundary-test reference was built by concatenating the terminal 5 kb of the plasmid, the complete plasmid sequence and the initial 5 kb of the same plasmid, thereby converting the circular junction into a linear region [58, 59]. ONT reads from the corresponding isolate were mapped to these references with minimap2 (map-ont preset) and sorted/indexed with SAMtools v1.2 [39]; reads with mapping quality ≥20 whose alignments crossed either artificial boundary were counted as boundary-spanning reads, and median depth was calculated in ±500 bp windows around both boundaries. This provided direct long-read support for circular continuity of all four reconstructed plasmids. Self-blast terminal-overlap screening was negative for all plasmids after removal of full-length self-hits (consistent with overlap trimming during reconstruction), and the MOB-suite circularity_status field was ‘not tested’ in this run; neither was used as circularization evidence. Full plasmid typing and validation metrics are provided in Table S6 and plasmid maps are provided in supplementary File-1.

### Phylogenomic and comparative genome analysis

To obtain a wider national-scale perspective, all *E. coli* genomes from Pakistan carrying *tet*(X4) and/or *mcr-1.1* were retrieved from the NCBI Pathogen Detection database. Only complete or potentially clean assemblies were retained after curation, yielding 213 publicly available genomes; combined with the 18 genomes generated in this study, the final comparative dataset comprised 231 genomes. Metadata, including collection year and source, were extracted with the Bio.Entrez module of Biopython (https://github.com/biopython/biopython). The workflow described above was then applied to the combined dataset. For phylogenomic analysis, all assemblies were processed with PPanGGOLiN v2.2.2 [60], to infer core genome and defined gene families present in ≥90% of genomes (3,454 families). The corresponding concatenated amino-acid alignment comprised 1,121,151 sites across the 231 genomes, of which 93.1% were constant and 46,566 were parsimony-informative (170,590 distinct site patterns). A maximum-likelihood tree was reconstructed with IQ-TREE v2.4.0 [61] under the best-fit substitution model LG+I+G4 (model-defined amino acid frequencies; proportion of invariable sites=0.875; gamma shape α=0.505 across four rate categories), with branch support estimated from 1,000 ultrafast bootstrap (UFBoot2) replicates [62], and the visualizations and annotations of the tree were generated using iTOL [63].

Phylogroups were determined with EZClermont [64], and serotyping profiling was performed with ECTyper [65]. All assemblies were additionally screened for adhesion-associated genes using the adhesiomeR package in R [66] shown in Table S9 and Fig. S3. The genomic context of *mcr-1.1* and *tet*(X4) was explored with Flankophile v0.2.9 [67] within a Snakemake v6.9.1 [68] workflow, and the flanking regions were reconstructed and visualized using Clinker v1.32 [69] and gggenes [70] together with custom R scripts. Fossilization of *mcr-1.1* was assessed using mcroni (https://github.com/liampshaw/mcroni). Full mcroni outputs are given in Table S12.

## Results

### Prevalence of *mcr-1.1* and *tet*(X4) across production stages

From 200 samples collected across four production stages of an integrated broiler production system, 18 *E. coli* isolates were recovered on colistin or tigecycline supplemented media, yielding an overall recovery rate of 9.0% (18/200). Stage-wise, positive isolates were recovered from broiler breeders (7/50, 14.0%), day-30 broilers (8/50, 16.0%), day-1 chicks (1/50, 2.0%) and retail chicken meat (2/50, 4.0%), demonstrating the presence of last-resort resistance determinants at every stage of the production continuum. All 18 phenotypically resistant isolates were confirmed by conventional PCR, demonstrating 100% concordance between phenotypic resistance and the presence of the corresponding genetic determinant. *E. coli* isolate SAMN48080415, recovered from a broiler breeder, harboured both *mcr-1.1* and *tet*(X4) simultaneously.

Plasmid-mediated *tet*(X4) gene was the predominant resistance determinant, detected in 11 of 18 isolates (61.1%). It was most prevalent among broiler breeders (5/7, 71.4%) and day-30 broilers (5/8, 62.5%), one isolate from retail meat (1/2, 50.0%). *mcr-1.1* was detected in 7 of 18 isolates (38.9%), being identified at all four stages: breeders (1/7, 14.3%), day-1 chicks (1/1, 100%), day-30 broilers (3/8, 37.5%) and retail chicken meat (1/2, 50.0%).

### Phylogenomic diversity and clonal transmission across poultry production sector

**Fig. 1.**
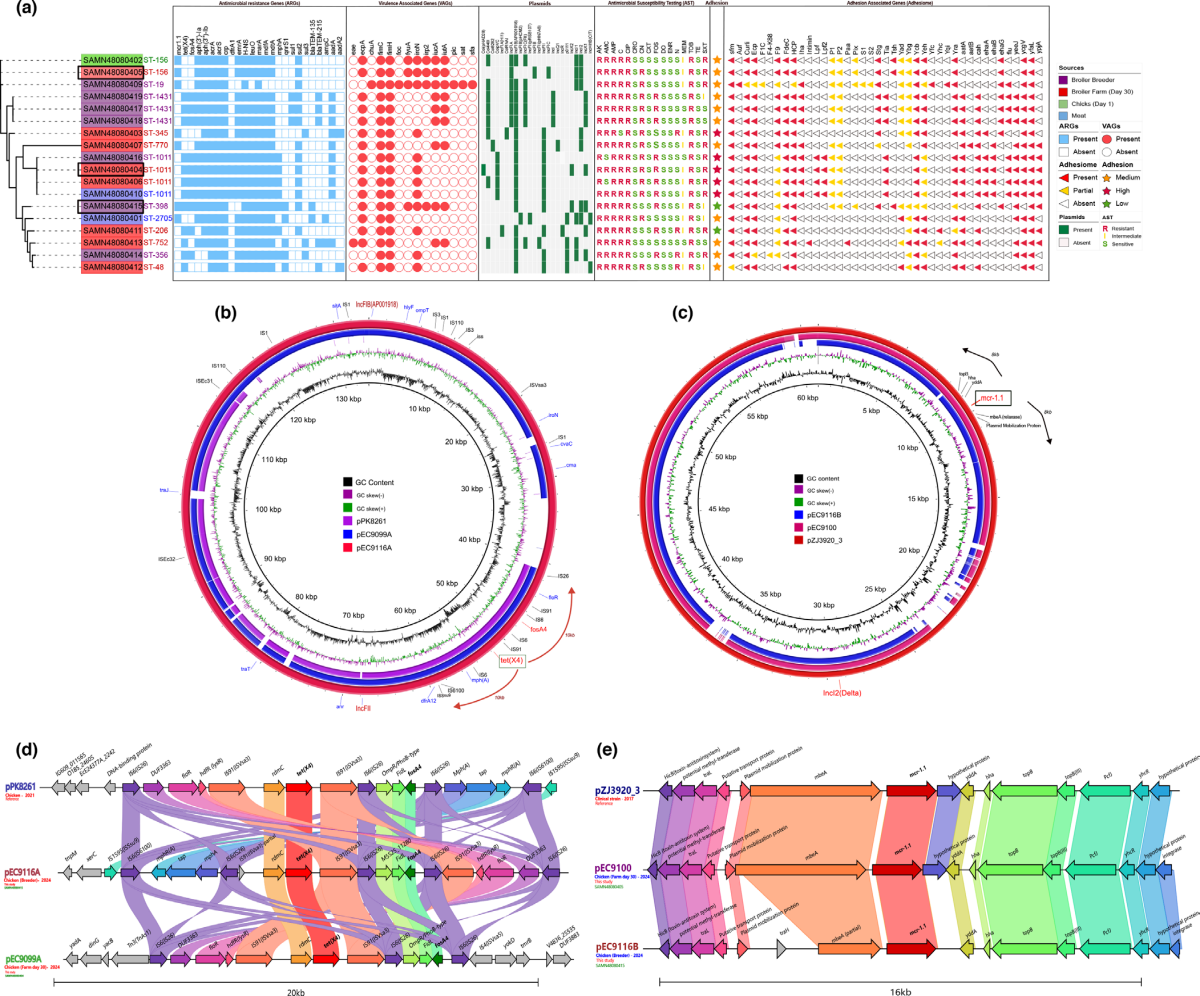
Phylogenomic structure and genetic context of *tet*(X4) and *mcr-1.1* from farm-to-fork isolates. (**a**) Represents the core-genome phylogeny of the *E. coli* isolates in this study, visualized by iTOL and annotated with multilocus STs, ARGs, VAGs, plasmid replicon types, phenotypic data from antimicrobial susceptibility testing (AST) profiling, results of the adhesion assays and adhesion loci (adhesiome). (**b**) and (**c**) depict the comparative alignment of plasmids resolved using long-read sequencing for *tet*(X4) and *mcr-1.1,* respectively, visualized by BRIG. Isolates designated for long-read sequencing are indicated in the tree with squared outlines. (**d**) and (**e**) represent comprehensive gene neighbourhood analyses generated with Clinker for the resistance loci (*tet*(X4)±10 kb and *mcr-1.1*±8 kb upstream and downstream, respectively) on the same long-read-resolved plasmids as shown in (b) and (c), showing local synteny, gene orientation, homology and MGEs.

Core-genome phylogenetic analysis of the 18 *mcr-1.1*- and/or *tet*(X4)-positive *E. coli* isolates revealed a polyclonal population structure, with isolates distributed across 12 distinct STs resolving into two major clades (Fig. 1a). Clade 1 comprised eight isolates belonging to five STs: ST156 (*n*=2), ST19, ST1431 (*n*=3), ST345 and ST770. This clade was characterized by predominance of *mcr-1.1* carried on conserved IncI2 plasmids. Within this clade, the three ST1431 isolates (SAMN48080417, SAMN48080418, SAMN48080419) formed a tight sub-cluster, all recovered exclusively from broiler breeders and carrying *tet*(X4), suggesting a localized clonal persistence of this lineage at the breeder level. Clade 2 consisted of ten isolates across seven STs, dominated by a clonal cluster of ST1011 (*n*=4), alongside ST398, ST2705, ST206, ST752, ST356 and ST48 (*n*=1 each). This clade was dominated by *tet*(X4) carriage on large IncFIB(AP001918)-IncFII megaplasmids. Within this clade, ST1011 was represented by four isolates spanning three sequential production stages, i.e. a broiler breeder, two day-30 broilers and a retail meat isolate, forming a well-supported sub-cluster consistent with clonal farm-to-fork transmission of a single *tet*(X4)-carrying lineage from the breeder flock through to the point of retail sale. Notably, the *E. coli* SAMN48080415 (ST398) isolate carrying both *mcr-1.1* and *tet*(X4) also resolved within this clade.

### Antimicrobial susceptibility profiling

Multidrug resistance was widespread based on the results of antimicrobial susceptibility testing for 18 isolates using 16 antimicrobials other than colistin and tigecycline that represent major classes (Fig. 1a). Ampicillin, amikacin, tobramycin and ciprofloxacin showed complete (100%) resistance. Additionally, high resistance was noted for chloramphenicol (94.4%), gentamicin (77.8%), amoxicillin-clavulanic acid (83.3%), trimethoprim-sulfamethoxazole (55.6% resistant, 22.2% intermediate), neomycin (22.2%) and FOS (50.0%). On the other hand, 100% susceptibility was noted for tetracycline, doxycycline, cefotaxime, ceftriaxone and enrofloxacin in all isolates. Meropenem was effective against all isolates, but 72.2% showed intermediate MICs.

### VAGs profiling

All isolates harboured a diverse array of VAGs (Fig. 1a), indicating pathogenic potential. Iron acquisition systems including *fyuA*, *iutA*, *iucA* and *iroN* were broadly distributed across both clades, reflecting the competitive fitness advantage conferred by siderophore-mediated iron scavenging in the intestinal environment. Type 1 fimbriae genes (*fimC*, *fimH*) were present in the majority of isolates, supporting adhesion capacity to epithelial surfaces. The adhesin gene *eae*, encoding intimin was also detected. Additional virulence determinants including *ecpA*, *chuA*, *pic* and *sat* were variably distributed, reflecting the diverse pathotypic backgrounds. Notably, isolate SAMN48080409 (Broiler-breeder with *mcr-1.1* resistance) with ST-19 was found to be harbouring the most VAGs, such as *chuA, foc, pic, sat and sfa* (haem utilization, F1-C fimbriae, serine protease, autotransporter toxin and S fimbriae, respectively), gene markers commonly associated with lineages like extraintestinal pathogenic *E. coli* (ExPEC) and uropathogenic *E. coli*.

### Biofilm, adhesion phenotypes and adhesiome

Biofilm assays yielded negative results across all test replicates in all three media. Based on the results of the adhesion assays on CHIC-8E11 cell line, all the isolates were classified into three groups depending on the adhesion rates, i.e. medium (27.8%), high (61.1%) and low (11.1%) as shown in Fig. 1a. High adhesion rate were depicted in 27.8% (5/18) (SAMN48080403, SAMN48080404, SAMN48080406, SAMN48080410, SAMN48080416), medium in 61.1% (11/18) and low in 11.1% (2/18; SAMN48080411, SAMN48080415). It is worth noting that all four ST-1011 isolates of meat samples had either high (3/4) or medium (1/4) adhesion phenotypes, indicating that this lineage has an increased colonization potential in food chain persistence and possible human exposure.

Heterogeneous distribution among the isolates was seen in all 41 loci encoding adhesins. All genomes preserved core adhesins such as *curli* fimbriae, *E. coli* common pilus *ecp*, type 1 fimbriae *sfm* and haemorrhagic coli pilus *HCP* exhibiting 100% presence partial or complete implicating in colonization and biofilm formation. There was stronger adhesion that was linked to certain loci. Long polar fimbriae were confined to high-adhesion isolates and the Tia invasin was predominantly found in high or medium adhesion isolates. Two thirds of the isolates contained P fimbrial operons partially. Presence of several adhesins loci showed consistent association with adhesion phenotypes. The long polar fimbriae system (Lpf/Lpf2), which is linked to intestinal persistence, was found in five isolates exhibiting high adhesion (SAMN48080404, SAMN48080406, SAMN48080410, SAMN48080416 from ST-1011; SAMN48080403 from ST-345). Similarly, the *Tia* invasin, mediating the invasion of epithelial cells, was found in 33.3% of isolates, mostly with medium-to-high profiles. The P fimbrial operons (*P1* and *P2*), which are generally associated with uropathogenic hierarchy, were partially present in 66.7% of isolates.

In context of autotransporters, *ehaA* and *ehaB* were identified in 33.3% and 27.8% of the isolates, respectively, albeit *ehaG* was reported in 50% isolates. *flu* gene responsible for encoding antigen 43, which facilitates biofilm formation and aggregation was identified in 77.8% of the isolates. Some of the adhesins such as, *ycgV, yeeJ, yfaL, ypjA*, and *cah*, did not strictly correlate with adhesion phenotypes and showed highly variable patterns.

### Plasmid replicon diversity

A total of 26 different replicon types across 18 isolates were identified, reflecting complete plasmidome architecture characterizing MDR *E. coli* isolates (Fig. 1a). IncFIB(AP001918) was most prevalent, detected in 88.9% of isolates followed by IncFII (66.7%), IncX1 (44.4%) and Col440I (44.4%). Replicons associated with *tet*(X4) and *mcr-1.1* resistance genes showed specific distribution patterns, e.g. IncFII replicons harbouring *tet*(X4) were detected in 66.7% of the isolates and IncI2 was detected in 38.9%. The *mcr-1.1*-associated IncI2 and *tet*(X4)-associated IncF plasmid clusters were classified by MOB-suite as having conjugative potential because both relaxase and MPF markers were detected (Table S13). IncX1 and IncX2 replicons were detected in 44.4 and 11.1%, respectively, which is commonly associated with quinolone resistance. IncHI1B(CIT) was detected in 11.1% and is mostly associated with clinical settings. Different col types (ColpVC, Col8282, Col(pHAD28) and ColRNAI) were distributed variably likely representing small plasmids assisting in accessory gene dissemination.

### Population genomic analysis *tet*(X4) and *mcr-1.1* across *E. coli* isolates (2011–**2024**)

**Fig. 2.**
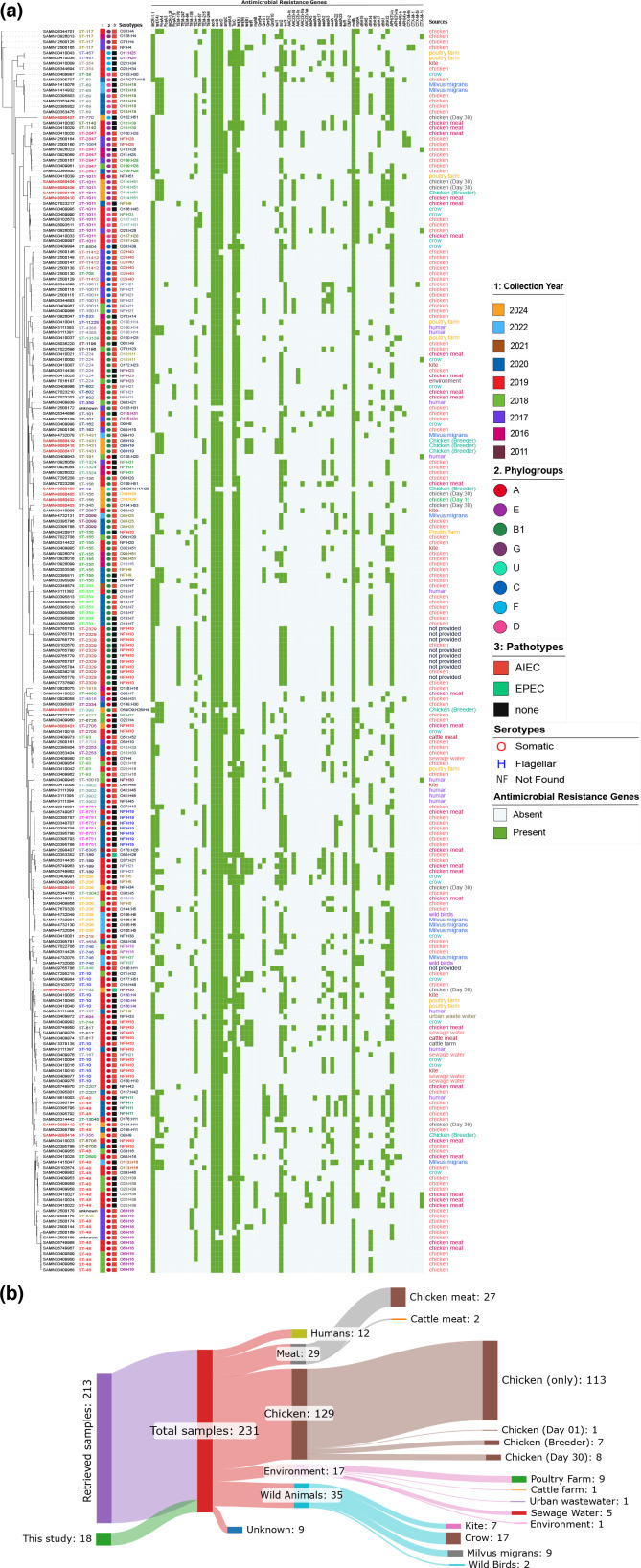
Population phylogenomic context of *tet*(X4) and *mcr-1.1* across 231 *E. coli* genomes and uniform source stratification. (**a**) Core-genome phylogenetic tree of 231 genomes (18 from this study, highlighted in red font, and 213 publicly available genomes) inferred using IQ-tree with 1,000 bootstrap replicates and visualized in iTOL. Annotation tracks depict STs, collection year, phylogroups, pathotypes, serotypes and ARGs. (**b**) Sankey diagram summarizes the source stratification used for downstream analyses (ARG stress and MGE analysis across niches), collapsing heterogeneous metadata into five major categories: chicken, environment, human, wild animals and meat.

In order to explore farm-to-fork transmission of AMR and specifically *mcr-1.1* and *tet*(X4) within the broader perspective, the 18 isolates were integrated with 213 publicly available *E. coli* isolates from Pakistan yielding the total dataset of 231 genomes from 2011 to 2024 (Fig. 2a).

The *mcr-1.1* gene was present in 192/231 of the genomes and the timeline shows that *mcr-1.1* was first isolated, analysed and published in 2016 [71]. However, according to the NCBI database, a previously isolated *mcr-1.1* encoding *E. coli* with BioSample number SAMN12098407 was collected in 2011 but submitted in 2019. Overall prevalence remained consistently high between 2017 and 2019, and detection continued throughout 2024. The source-based distribution across different reservoirs is shown in Fig. 2b: chicken sources (79.8%; 103/129), chicken meat (93.1%; 27/29), wild animals (71.4%; 25/35), human (100.0%; 12/12), environmental samples (94.1%; 16/17) and unknown sources (100%; 9/9) (Fig. 2b). Tigecycline resistance gene *tet*(X4) was reported in 75/231 genomes, representing a relatively new resistance mechanism with the earliest isolates being recovered in 2018 (*n*=3). Source-based prevalence pattern included chicken (41.1%; 53/129), chicken meat (13.8%; 4/29), human (33.3%; 4/12) and environmental isolates (17.6%; 3/17), with notably higher prevalence in *Milvus migrans* and wild birds (100%; 11/11), indicating disproportionate spread when compared with human isolates carrying *mcr-1.1*.

**Fig. 3.**
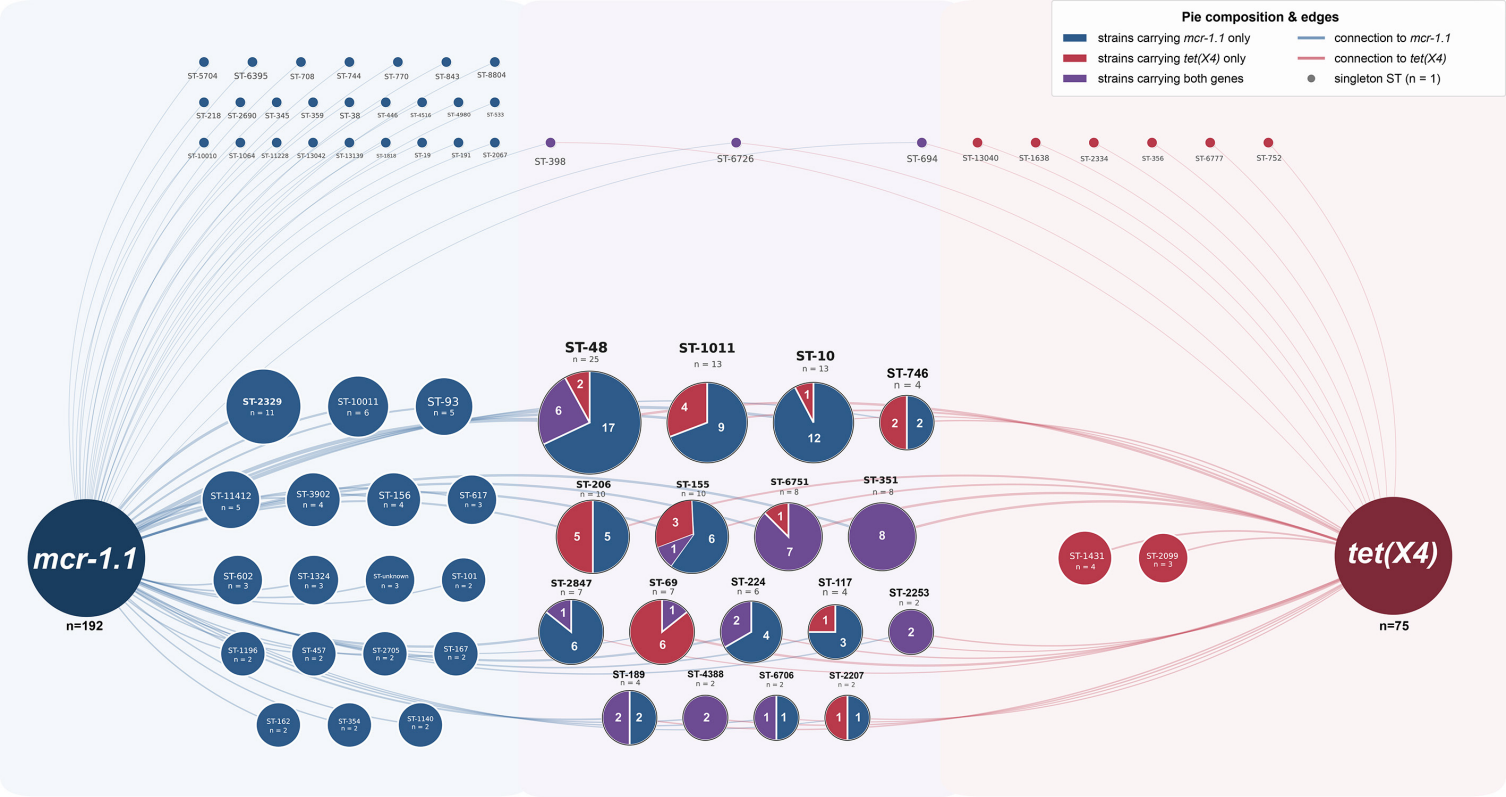
STs across *tet*(X4) and *mcr-1.1* harbouring genomes.

The data revealed 15.58% (36/231) isolates co-harbouring *tet*(X4) and *mcr-1.1*, depicting convergence of resistance to two last-resort antibiotics (Fig. 2a). This phenomenon was first observed in the isolate (SAMN17016187) collected in 2018 from environment, followed by the highest reports from 2020 (*n*=22), mainly in chicken. ST distribution in dual-positive isolates was dominated by ST-48 (*n*=6), ST-351 (*n*=8) and ST-6751 (*n*=7) (Fig. 3). Long-read sequencing of the single 2024 isolate (SAMN48080415, this study) showed that the two genes were carried by distinct plasmids; *tet*(X4) on a 134 kb IncFIB-IncFII megaplasmid (pEC9116A) and *mcr-1.1* on a second 63.6 kb IncI2 plasmid (pEC9116B).

### Molecular characterization (phylogroups, pathotypes and serotypes)

Clermont phylogroup classification revealed predominance of commensal-associated lineages (Fig. 2a). Phylogroup A was the most dominant at 48.5%, followed by phylogroup B1 at 28.1%, encompassing both commensal and diarrhoeagenic strains. Phylogroups associated with ExPEC, i.e. C, D, E, F and G, depicted decreasing frequencies in order of 5.6, 6.9, 6.5, 2.2 and 1.7%, respectively. A single isolate SAMN48080409 (Broiler breeder resistant to *mcr-1.1*) with ST-19 was allocated in phylogroup U, encoded extensive ExPEC virulence markers. Phylogroups distribution also varied with respect to the source categories. Pathotyping revealed Adherent invasive *E. coli* (AIEC) as the most dominant pathotype with 55.4% of the isolates and only two isolates were positive for EPEC, one of which is from this study belonging to ST-752 (day-30 broiler) (Fig. 1a). Remaining isolates lacked definitive pathotyping markers but still carried VAGs and adhesins.

The serotype analysis showed significant antigenic heterogeneity among the 231 genomes, with more than 80 different O:H combinations observed (Fig. 2a). Various isolates had non-typeable (NF) O-antigens, indicating new or insufficiently described somatic antigen forms, whereas H-antigen determination was more extensive. ST-1431 breeder isolates (SAMN48080417, SAMN48080418 and SAMN48080419) were of identical serotype O8:H19, which is in line with the observation of clonal expansion of the breeder flock. ST-1011 isolates on day-30 broilers and meat (SAMN48080404, SAMN48080406, SAMN48080410 and SAMN48080416) were serotype O114:H51. ST-156 isolates that were harbouring *mcr-1.1* (SAMN48080402 and SAMN48080405) were identified as serotype O54:H28. Serotype O15:H18, which is associated with UTIs in humans, was detected in chicken isolates (*n*=4) and a single *M. migrans* isolate. O18:H7 linked to neonatal meningitis was detected in the chicken (*n*=6) and human (*n*=1) isolates, indicating that clinically relevant serotypes have potential zoonotic routes of transmission.

### Sequence types

Multilocus sequence typing identified 87 distinct STs across the 231 genomes, revealing extensive genetic diversity underlying resistance gene carriage (Fig. 2a) and substantial overlap, indicating that both resistance genes disseminate through the same successful clonal backgrounds (Fig. 3). The most prevalent STs reported across all isolates were, i.e. ST-48 (*n*=31; 13.4%), ST-10 (*n*=13; 5.6%), ST-155 (*n*=11; 4.8%), ST-206 (*n*=10; 4.3%), ST-1011 (*n*=13; 5.6%), ST-351 (*n*=8; 3.5%), ST-2329 (*n*=10; 4.3%) and ST-6751 (*n*=15; 6.5%). These STs are globally distributed and frequently associated with AMR in livestock and clinical settings. ST-48, the single most prevalent lineage, demonstrated remarkable temporal persistence and ecological spread. ST-48 isolates were recovered from 2016 through 2024, across chicken, chicken meat, *M. migrans*, human and environmental sources, carrying both *mcr-1.1* (*n*=23) and *tet*(X4) (*n*=8), including five isolates with dual carriage. This versatile lineage appears to function as a ‘super transmitter’ ST, facilitating resistance gene exchange across ecological classes. ST-1011, the dominant lineage in our farm-to-fork study, showed expanding representation over time: first detected in 2016–2017 (*n*=4), with increasing prevalence through 2019 (*n*=4), and continued presence in our 2024 sampling (*n*=4). All ST-1011 isolates from 2024 (this study) were *tet*(X4)-positive but *mcr-1.1*-negative, while earlier ST-1011 isolates (2016–2019) predominantly carried *mcr-1.1* with variable *tet*(X4) status. This temporal shift suggests evolving plasmid dynamics within this successful lineage.

Twenty STs harboured strains carrying *mcr-1.1* and *tet*(X4) across either the same or different isolates, comprising 56.3% (130/231) indicating that the two resistance genes converge on a relatively small set of STs (Fig. 3). The remaining isolates were distributed across 43 *mcr-1.1* exclusive STs (*n*=88) and eight *tet*(X4) exclusive STs (*n*=13). Co-occurring STs did not necessarily reflect co-carriage within individual isolates but rather indicate that identical genetic backgrounds independently support one or both resistance determinants (Fig. 3).

Plot summarizes the set of STs observed among genomes carrying *tet*(X4) and *mcr-1.1*. The middle region depicts the ST categories that are present in both groups (i.e. same STs observed among *tet*(X4) and in *mcr-1.1*).

Lineage-level patterns revealed marked asymmetry in resistance association. ST-48 showed strong enrichment for *mcr-1.1* relative to *tet*(X4), consistent with preferential compatibility with IncI2 plasmids, while still supporting *tet*(X4)-carrying IncF plasmids in a subset of strains. ST-10 displayed a similar bias toward *mcr-1.1* with minimal *tet*(X4) presence, suggesting limited plasmid compatibility or delayed *tet*(X4) entry following its recent emergence. In contrast, ST-351 exhibited a balanced distribution with frequent dual carriage, indicating a high propensity for accumulating multiple resistance plasmids. ST-1011 showed a temporal shift, with earlier isolates carrying *mcr-1.1* and recent farm-to-fork isolates exclusively harbouring *tet*(X4), consistent with plasmid replacement or changing selective pressures. ST-206 showed equal but largely independent acquisition of both genes, whereas ST-69 was strongly associated with *tet*(X4), particularly among *M. migrans* isolates, suggesting a role for wild birds in disseminating this ST–gene combination.

### Plasmid architecture and resistance gene localization

To resolve complete plasmidomes, long-read sequencing on three representative isolates was carried out (highlighted with black outline Fig. 1a), with resistance target genes *mcr-1.1* and *tet*(X4). Full plasmid characterization was done with typing and stratified AMR profiles as shown in Table 1. The dual-positive breeder isolate SAMN48080415 (ST-398) had *tet*(X4) on pEC9116A (134 kb, IncFIB-IncFII) and *mcr-1.1* on pEC9116B (63.6 kb, IncI2), separate conjugative plasmids that allow independent dissemination (Fig. 1b and 1c). Day-30 isolate SAMN48080404 (ST-1011) harboured *tet*(X4) on pEC9099A (148 kb, IncFIB-IncFII), whereas day-30 isolate SAMN48080405 (ST-156) carried *mcr-1.1* on pEC9100 (58.6 kb, IncI2).

**Table 1.** Genomic characterization of three strains by hybrid assembly approach

Strain ID	Component	Size	AMR gene
**SAMN48080415**	**Chromosome**	~5.00 Mb	*mdtF, mdtE, gadX, gadW, msbA, marA, ampC, ampC1, mdfA, acrA, acrS, acrE, acrF, pmrF, bacA, ramA, emrR, emrB, emrK, emrE, yojI, evgS, evgA, baeR, baeS, cpxA, H-NS, CRP, ampH*
pEC9116A	**IncF megaplasmid** **[IncFIB(AP001918)-IncFII]**	134 kb	*fosA4, mph(A), floR, **tet***(X4)*, dfrA12*
pEC9116B	**IncI2 plasmid**	55.1 kb	***mcr-1.1***
	**IncX1 plasmid**	48.5 kb	*aph(3')-Ia, blaTEM-1B, blaTEM-106, floR*
	**IncX2 plasmid**	7.2 kb	*qnrS13, tet(A*)
	**Cryptic components**	~7 kb	*aph(6)-Id, aph(3'')-Ib*
**SAMN48080404**	**Chromosome**	~5.07 Mb	*mdtF, mdtE, gadX, gadW, msbA, marA, ampC, ampC1, mdfA, acrA, acrS, acrE, acrF, pmrF, bacA, ramA, emrR, emrB, emrK, emrE, yojI, evgS, evgA, baeR, baeS, cpxA, H-NS, CRP, ampH, ugd, kdpE*
pEC9099A	**IncF megaplasmid [IncFIB(AP001918)-IncFII]**	148 kb	*mph(A), dfrA12, AAC(3)-IId, fosA4, floR, **tet***(X4)
	**IncX1**	44 kb	*aph(3')-Ia, blaTEM-1B, blaTEM-106, floR*
	**IncX2**	19 kb	*qnrS13, tet(A*)
	**ColpVC**	3.9 kb	None
	**Col(pHAD28**)	6.4 kb	None
	**Cryptic components**	<10 kb	*aph(6)-Id, aph(3'')-Ib*
**SAMN48080405**	**Chromosome**	~4.95 Mb	*mdtF, mdtE, gadX, gadW, msbA, marA, ampC, ampC1, mdfA, acrA, acrS, acrE, acrF, pmrF, bacA, emrR, emrB, emrK, emrE, yojI, evgS, evgA, baeR, baeS, cpxA, H-NS, CRP, ampH, kdpE*
	**IncF megaplasmid [IncGIA, IncFIB, IncFIC]-rep 2244**	216 kb	*aph(6)-Id, aph(3'')-Ib, aph(3')-Ia, sul2, tet(A), floR*
pEC9100	**IncI2**	60.8 kb	***mcr-1.1***
	**IncI1(alpha**)	23.5 kb	None detected
	**Cryptic components**	<10 kb	*blaTEM-1B, mphB*

BRIG analysis revealed great structural similarity of the study and reference plasmids. The *tet*(X4) carrying plasmids were similar to 119 kb IncFIB-IncFII plasmid of Pakistani origin chicken sample from 2021, pPK80156 as reference, and to the 61 kb IncI2-based plasmid of a clinical isolate collected in 2017 from China pZJ3920_3 carrying *mcr-1.1*. The stability of plasmid architecture in breeder and day-30 isolates and 2017 clinical vs 2024 poultry sources indicate endemic circulation of resistance plasmids throughout the production system and possible clinical interface (Fig. 1b and c).

**Fig. 4.**
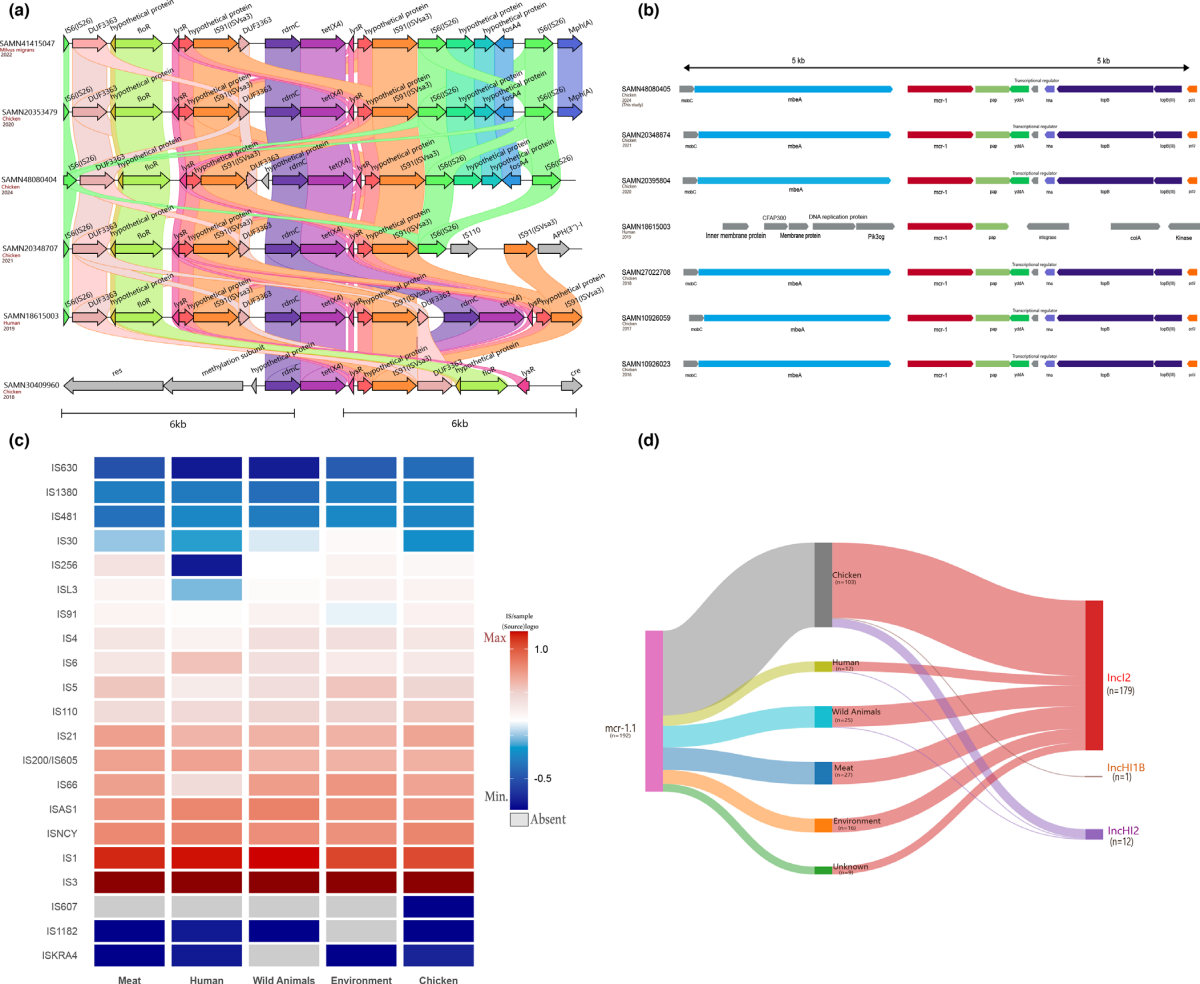
MGEs and insertion sequence (IS) profiles associated with *mcr-1.1* and *tet*(X4) across one health sources. (**a**) and (**b**) depict comparison of *tet*(X4) and *mcr-1.1* genomic neighbourhood from different sources integrated with samples from this study across different years, constructed through clinker and gggenes, respectively. The resistance locus is embedded within a cassette depicted with different arrows and colours for different determinants in the upstream and downstream flanks of the target genes. Gene arrows indicate orientation and functional annotation. Homology ribbons denote gene conservation across different sources and collection years. (**c**) Heatmap summarizing the insertion sequence (IS) family densities (mean copies per sample) across five categories. Colour intensities depict IS density as described in legend; rows and columns are hierarchically clustered. (**d**) Sankey diagram showing the distribution of *mcr-1.1* positive strains across different sources into different plasmid backgrounds.

Plasmid replicon typing of 192 *mcr-1.1* carrying isolates found majorly IncI2 (93.2%, 179/192) involvement with minor IncHI2 (6.25%) and IncHI1B (0.5%) (Fig. 4d). Among all sources, IncI2 was most dominant in chicken, followed by meat, human and wild animals, indicating one common transmission network based on poultry production. It was found that the 18 farm-to-fork isolates from this study harboured 26 unique plasmid replicons with IncFIB(AP001918) as the most common, followed by IncFII and IncI2.

### Contrasting evolutionary trajectories of *mcr-1.1* and *tet*(X4) MGEs

Comparative study of the flanking regions showed that there were essentially different evolutionary states of the two resistance determinants (as shown in Fig. 1d, e and Fig. 4a, b). The *tet*(X4) cassette possesses the full mobility potential of a nested transposon structure, i.e. the outer boundaries of the transposons IS26 created a pseudo-compound transposon, with inner IS91/ISVsa3 elements in direct contact with *tet*(X4) and allowing rolling-circle transposition. This architecture expressed three resistance genes together as a multi-resistance module: *tet*(X4) (tigecycline), *floR* (florfenicol) and *fosA4* (FOS) with any one antimicrobial co-selecting the other two resistance genes. Structural changes between breeder (SAMN48080415, pEC9116A, inverted orientation) and day-30 (SAMN48080404, pEC9099A, canonical orientation) plasmids indicates recombination of the IS26 occurrence during transmission but the content of the core resistance was retained.

In case of *mcr-1.1*, it has become evolutionarily stabilized as demonstrated by the absence of canonical mobilizing element, ISApl1 previously responsible for mobility, was not present in any of the 192 positive isolates. The flanking structure (*mobC-mbeA-mcr-1.1-pap2-yddA-hha-topB-pcfJ*) was stable in chicken isolates, suggesting post-mobilization stabilization in IncI2 plasmid backbones (Fig. 4b). This confirms that transmission occurs entirely through plasmid conjugation after acquisition of the gene through Tn6330 (ISApI1 transposition). It is also important to note that the human clinical isolate from 2019 had a unique genetic context with a gene encoding integrase enzyme downstream of *mcr-1.1*, which was not found in any of the poultry isolates.

A broader range of 21 IS families with source-specific patterns were identified in the IS element profiling across sources (Fig. 4c). Human isolates had higher density of IS6 with the log_10_ values ranges and dramatically less IS256 which may be an indication of different forms of selection in the clinical and agricultural setting. The density of the IS91 was similar between chicken and wild animals which is also consistent with active *tet*(X4) element transfer between those two groups.

### AMR and VAGs across differential variables in the population

**Fig. 5.**
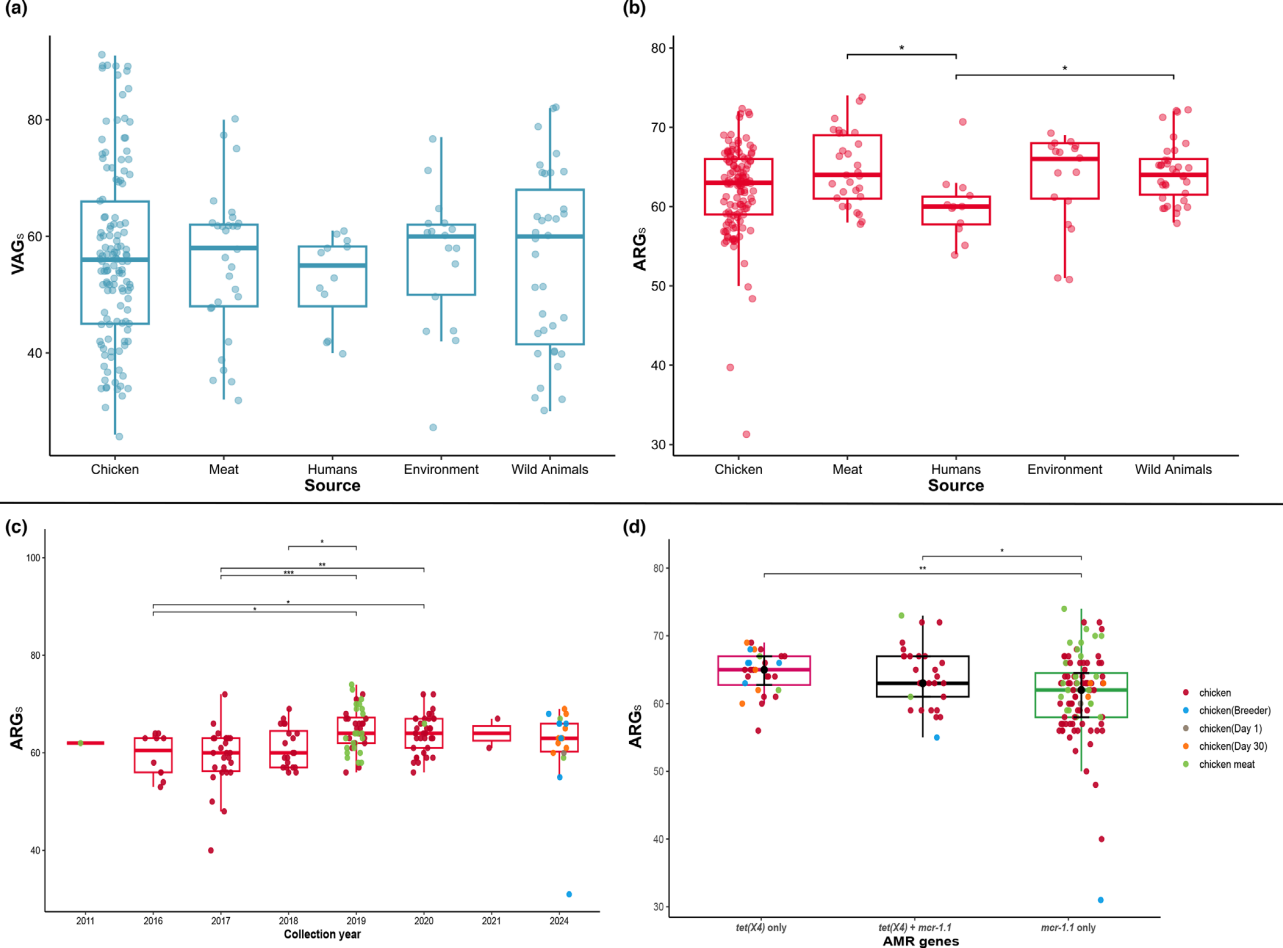
ARG and VAG burden patterns across time, sources and stratification across *mcr-1.1* and *tet*(X4). (**a**) VAG counts across source categories (**b**) ARG counts across source categories. (**c**) Temporal ARG burden in chicken isolates (2016–2024) only. (**d**) ARG burden stratified by resistance gene carriage in chicken only. Pairwise Wilcoxon Rank sum test with Benjamini-Hochberg correction (Table S8); *P_adj_<0.05, **P_adj_<0.01, ***P_adj_<0.001. The boxplot centre line denotes the median, the box limits denote the first and third quartiles (interquartile range, IQR) and the whiskers extend to the most extreme values within 1.5×IQR of the box; individual points represent single genomes. Source-group sizes are listed in Table S7.

The burden of VAG was homogenous across all categories of sources (*p_adj_*.>0.10 across all comparisons), showing that all sources exhibit equal pathogenic capacity regardless of their isolation origin (Fig. 5a). As a contrast, there was significantly high inter-source variation in ARG pattern.

The number of ARGs in human clinical isolates was significantly lower than in isolates from meat (*P_adj_*.=0.025) and wild animals (*P_adj_*=0.015), while no significant differences were observed among the remaining sources (Fig. 5b). Temporal analysis of chicken isolates showed that there is a significant increase in ARG burden: 2017 vs 2019 (*P_adj_*.=0.001), 2016 vs 2019 (*P_adj_*.=0.031) and 2017 vs 2020 (*P_adj_*.=0.004) (Fig. 5c). The 2024 isolates maintained this elevated level, indicating stable establishment of high burden. Resistance gene carriage stratification showed that *tet*(X4)-bearing isolates had much more ARG burden than *mcr-1.1*-only isolates (*P_adj_*.=0.005), whereas the difference in ARGs between dual-positive (*tet*(X4) *+ mcr*−1.1) and *tet*(X4)-only isolates was not significant (*P_adj_*.=0.446) (Fig. 5d).

## Discussion

The occurrence of resistance to last-resort antimicrobials like colistin and tigecycline in the poultry food chain is a significant menace to veterinary practice and to human health given the critical role of these agents [25, 72]. Although tigecycline is not used in poultry production, selective pressure from other commonly used antimicrobials can co-select plasmids carrying *tet*(X4), facilitating their persistence and spread via MGEs [28, 73]. This study presents extensive genomic evidence of farm-to-fork dissemination and spread of plasmid-mediated resistance against colistin (*mcr-1.1*) and tigecycline [*tet*(X4)] in poultry production systems from Pakistan revealing unique evolutionary trajectories and transmission dynamics for each gene. By integrating 18 farm-to-fork *E. coli* isolates with 213 publicly available genomes spanning 2011–2024 and different sources, we demonstrate that these critical ARGs have achieved endemic spread within a unified transmission network connecting breeders, day-old chicks, day-30 chickens, retail meat and then also spilling into wild birds, environment and clinical settings across Pakistan.

The polyclonal diversity observed across our farm-to-fork isolates, 12 different STs observed in 18 isolates strongly supports the argument of horizontal gene transfer as the dominant mechanism of dissemination rather than clonal expansion of a single lineage. This observation is aligned to the reported global trends in epidemiological patterns of *mcr-1.1*-positive *E. coli* in poultry production systems where clonal diversification has been reported [74, 75]. The fact that ST-1011 is the major lineage with clear evidence of farm-to-fork transmission suggests that some of the clonal backgrounds have the ability to continue across the production spectrum.

The prevalence of phylogroups A (44.2%) and B1 (31.6%) in all categories of sources can be correlated with their known presence as commensal lineages in poultry intestinal microbiota [76, 77]. Nevertheless, the simultaneous occurrence of ExPEC such as ST-69, ST-73, ST-95 and ST-131 in the broader data set suggests that poultry production systems harbour clinically significant pathogenic clones [78]. It is especially concerning that the most widespread is AIEC pathotype (55.4% of all isolates), as the AIEC strains have the ability to adhere to and invade intestinal epithelial cells [79]. The observation of consistent VAG burden of all types of sources (*P_adj_*>0.10 in all comparisons) points to the fact that poultry-derived *E. coli* share the same pathogenic potential as human clinical isolates speaks in favour of the zoonotic nature of the poultry-associated *E. coli* populations.

Our finding that ISApI1 was completely absent from all *mcr-1.1*-positive isolates across years of surveillance (2011–2024) provides compelling evidence for post-mobilization stabilization of colistin resistance within Pakistan’s poultry ecosystems. This observation aligns with the evolutionary model proposed by Snesrud and colleagues, who demonstrated that the ISApI1-mcr-1-ISApI1 composite transposon (Tn6330) has an intrinsic propensity to lose one or both flanking ISApI1 copies through abortive transposition, resulting in the loss of transposability and stabilization of the resistance gene within diverse plasmid backbones [80, 81]. Similar post-mobilization stabilization has been documented in European and Asian poultry populations, where ISApI1-deficient *mcr-1.1* structures are now predominant [82, 83].

The huge majority of IncI2 plasmids bearing *mcr-1.1* (93.23% among all sources) is in agreement with epidemiological trends in the rest of the world, where IncI2 is the most commonly reported plasmid incompatibility (Inc) group with *mcr-1.1* spread [84, 85]. IncI2 plasmids have a number of features that support effective spread: they are relatively small (~60 kb), thus minimizing the fitness costs; they transfer with high frequency in intestinal conditions; and they have a limited host range that includes some of the most important Enterobacteriaceae pathogens [86]. The high level of conserved IncI2 carriage in chicken (89.3%), human (91.67%), wild animal (96.0%), meat (100%) and environmental (100%) sources of data are strong arguments in favour of a transmission network, which is why this pattern coincides with the ecological connectivity model developed by Xu and colleagues that identified 195 plasmids that were shared by human, animal and environmental sectors in Hong Kong urban ecosystems [87].

Consistency in the *mcr-1.1* flanking region structure (*mobC-mbeA-mcr-1.1-pap2-yddA-hha-topB-pcfJ*) among chicken isolates of 2016–2024 also supports endemic circulation of certain plasmid lineages in the production system. Interestingly, one of the human isolates collected in 2019 [25] reported in a study in 2021, displayed a unique genetic background. Although, the original study did not examine the flanking cassette structure around *mcr-1.1*, our analysis shows specifically the genetic context. This revealed the presence of an integrase gene positioned downstream *mcr-1.1,* a feature that was not observed in any of the poultry-derived isolates analysed in our dataset. This structural divergence is indicative of the fact that food chain transmission does take place, as evidenced by similar STs between chicken and human sources, but that independent clinical acquisition events are also involved [88].

Our data show in stark contrast to the stabilized *mcr-1.1* that *tet*(X4) still has the potential of being mobile in actively evolving mobile genetic environments. A nested transposon structure has been identified where outer boundaries of IS26 make a pseudo-compound transposon with inner elements of IS91/ISVsa3 directly bordering *tet*(X4) gene, which is in line with the canonical architecture that follows the discovery of the plasmid-mediated tigecycline resistance in China in 2019 [28, 89]. The neighbouring ISCR2 or IS26 genes found upstream and downstream of *tet(X*) genes and their joint activity are key driving factors during the horizontal gene transfer and have been the main mechanisms of spread all over the world [90]. ISCR2 can effectively capture adjacent sequences without the use of terminal inverted repeats, constituting a typical rolling-circle transposable unit [91]. At the same time, IS26 contributes to plasmid fusion and recombination of genes by both conservative and replication transposition, which allows the *tet*(X4) element to reach a variety of plasmid replicons [92]. The plasmid architecture establishes co-selection dynamics in which the use of either one antimicrobial (tigecycline, florfenicol or FOS) in the production of poultry will be selective to all three antimicrobials – a phenomenon of special concern considering that florfenicol continues to be used in Pakistani veterinary practice [93].

Importantly, *tet*(X4)-positive *E. coli* and *Klebsiella pneumoniae* have been detected in black kites (*M. migrans*) in Pakistan, and whole-genome analysis has shown syntonical arrangements in genomic background of *tet*(X4) carrying plasmids in wild birds and poultry, providing molecular support for poultry to wild bird transmission in this niche [90, 94]. The black kites are the peridomesticated raptors and they are frequent visitors of poultry farms, slaughterhouses and urban waste areas, and this puts them in the position of possible agents of resistance gene transfer between the farming and environmental segments [26, 95]. The same density of IS91 in both chicken and wild bird isolates indicates that active element transfer is taking place between these populations and thus in agreement with the rolling-circle transposition mechanism utilized by ISCR2 to activate *tet*(X4) [96].

Our analysis depicts that *tet*(X4)-bearing isolates have much greater ARG burdens in comparison to *mcr-1.1*-only isolates (p_adj_=0.005). It is hypothesized that this is the result of plasmid architecture, with the *tet*(X4) locus found on IncF megaplasmids (134–148 kb) co-localizing numerous resistance genes (*floR, fosA4, mph(A), dfrA12*). Whereas *mcr-1.1* is found on relatively streamlined IncI2 plasmids (~60 kb) with less co-harbouring genes. Similar conclusions were drawn for *tet*(X4)-bearing plasmids in other countries; Du and colleagues described *tet*(X4) in 170 kb IncFIB plasmids in Chinese pigs with massive multidrug resistance regions with IS26 elements on either side [97], and Mohsin *et al*. reported *tet*(X4) in 113 kb IncFII plasmids in Pakistani poultry with a large number of resistance determinants [98]. Statistical confirmation of the nature of *tet*(X4) introduction in the AMR evolution of Pakistan’s poultry-associated *E. coli* strains is statistically supported by the large temporal change in ARG burden within chicken isolates coinciding with the rise of *tet*(X4) (2017 vs. 2019, p_adj_=0.001). The results of Lu and colleagues who identified *E. coli* isolates containing *tet*(X4), *mcr-1.1 and bla_NDM-5_* in Chinese pigeons showed that the dissemination of pan-resistant strains in animal production systems is clonal [99]. Cases of *tet*(X4) and bla_NDM-5_ co-occurrence on plasmids in multidrug-resistant *E. coli* of Chinese chicken were later reported by Sun and colleagues [100]. The presence of *bla_NDM_*-carrying isolates in the larger Pakistani dataset in which the *tet*(X4) or *mcr-1.1* genes are present is a good example of this intersection of resistance to multiple last-resort antibiotics [101, 102].

The integrated transmission network that is reported in this research: breeders, broilers, meat, wild birds and humans with common STs and plasmid profiles, is an example of the One-Health aspects of AMR. Cross-sectorial sharing of strains has also been reported elsewhere: in Hong Kong, Xu and colleagues found 142 instances of strain sharing between human-associated and environmental samples of AMR [87], and Li and colleagues found that ~40% of AMR genes were shared between *E. coli* from humans and chickens in Chinese poultry production systems, indicating substantial overlap in resistance profiles across the human and animal interface [103]. All these results indicate a need to adopt a multisectorial surveillance throughout the entire transmission network, with coordinated sampling procedures that allow direct comparison of human, animal and environmental divisions.

While the overall study has immense implications for global and national governance and policy to combat AMR dissemination in the food chain, several limitations should be noted. The primary sampling was restricted to a single vertically integrated broiler production within a specific time frame resulting in smaller sample size and limited geographical scope. Furthermore, to make conclusive arguments for the dissemination mechanisms of AMR to the customer level, future studies should incorporate detailed antimicrobial usage data and broader surveillance retail meat. Additionally, the potential for a cross-environmental transmission route involving wild birds is critical, but our sampling was confined to poultry. Therefore, future research should also focus on comprehensive sampling of surrounding wildlife and environmental vectors to map the direction and extent of this spillover.

## Conclusions

In this study, it is confirmed that *mcr-1.1* and *tet*(X4) spread through farm-to-fork poultry production through two fundamentally different mechanisms: *mcr-1.1* has become endemic-stable in IncI2 plasmid backbones following ISApl1 loss and spreads exclusively through plasmid conjugation and *tet*(X4) is actively mobile in IS26/ISCR2-bounded transposon-based transposons that are capable of capturing AMR genes. Clonal ST-1011 isolates were detected both at farm and retail levels, which provides molecular support of food chain transmission of tigecycline resistance up to the consumer level. Comprehensive country-wide analysis demonstrates that the phenomenon of conferring resistance to several last-resort antibiotics spurs the way for development of extensive drug resistance and pathogenic phenotypes. With few to no available treatment options leading, this signals the emergence of potential superbugs within the One-Health framework. This highlights an urgent need to conduct integrated surveillance across poultry production, retail meat, wild bird populations and clinical environments to track and curb the spread of last-resort AMR along the food chain.

## Supplementary material

10.1099/mgen.0.001800 Supplementary Material 1.

10.1099/mgen.0.001800 Supplementary Material 2.

## Abbreviations

AMR: antimicrobial resistance
ARG: antibiotic resistance gene
BRIG: blast Ring Image Generator
FOS: fosfomycin
LB: Luria–Bertani
MDR: multiple drug-resistant
MGE: mobile genetic element
MIC: minimum inhibitory concentration
ONT: Oxford Nanopore Technologies
ST: sequence type
VAG: virulence-associated gene
