# Supplementary material for: Integrated poultry production as a reservoir of tet(X4) and mcr-1.1 encoding Escherichia coli in Pakistan
Source: Microb Genom. 2026 Aug 3;12(8):001800. doi: 10.1099/mgen.0.001800 (PMC13431907; doi:10.1099/mgen.0.001800)
Supplement: Supplementary Material 1. [file mgen-12-01800-s001.pdf]

Integrated Poultry Production as a Reservoir of *tet(X4)* and *mcr-1.1* Encoding *Escherichia coli* in Pakistan

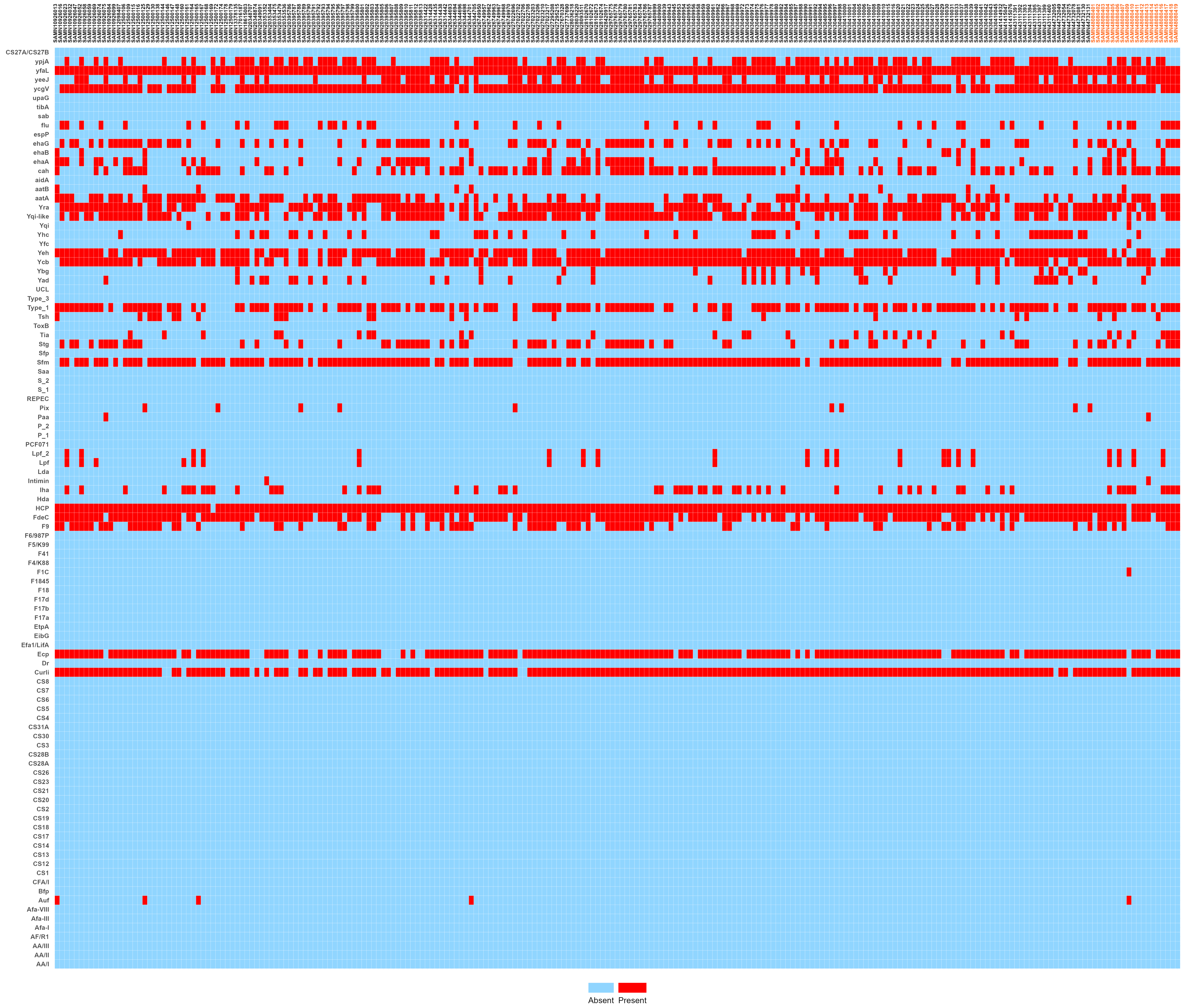

Supplementary Figure 1 depicting adhesiome found in 231 genomes. Red tiles: present, Blue tiles: Absent, visual depiction of table S9

Plasmid “pEC9099A” recovered from the strain: **SAMN48080404** harboring *tet(X4)*

Typing: IncF Megaplasmid [IncFIB(AP001918)-IncFII]

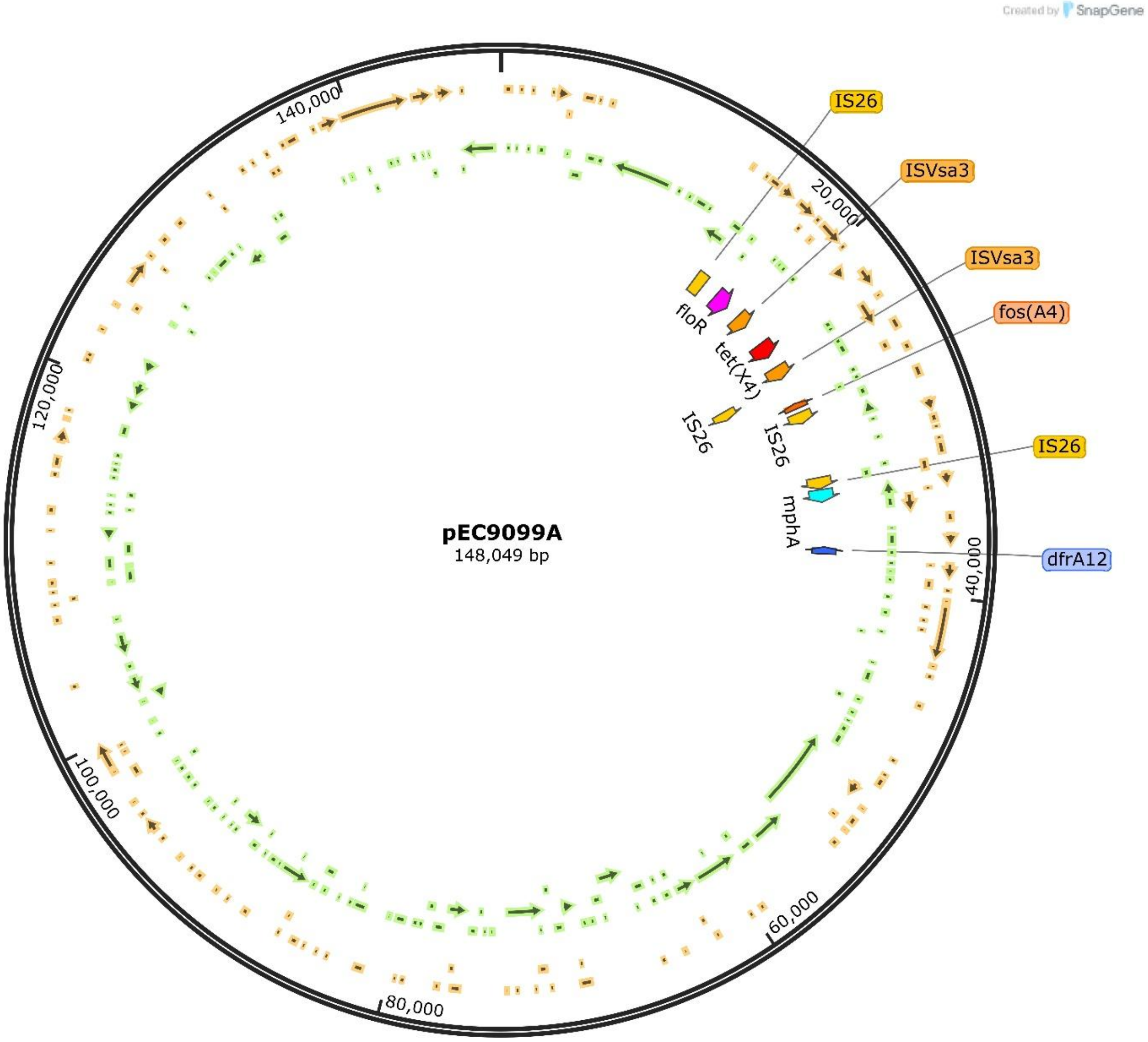

Supplementary Figure 2. Plasmid pEC9099A map showing the annotations for regions containing *tet(X4)* and *fos(A4)* with all related IS regions. Detailed segment annotation is depicted in Figure 1(B and D) and table 1.

Plasmid “pEC9100” recovered from the strain: **SAMN48080405** harboring *mcr-1.1*

Typing: IncI2

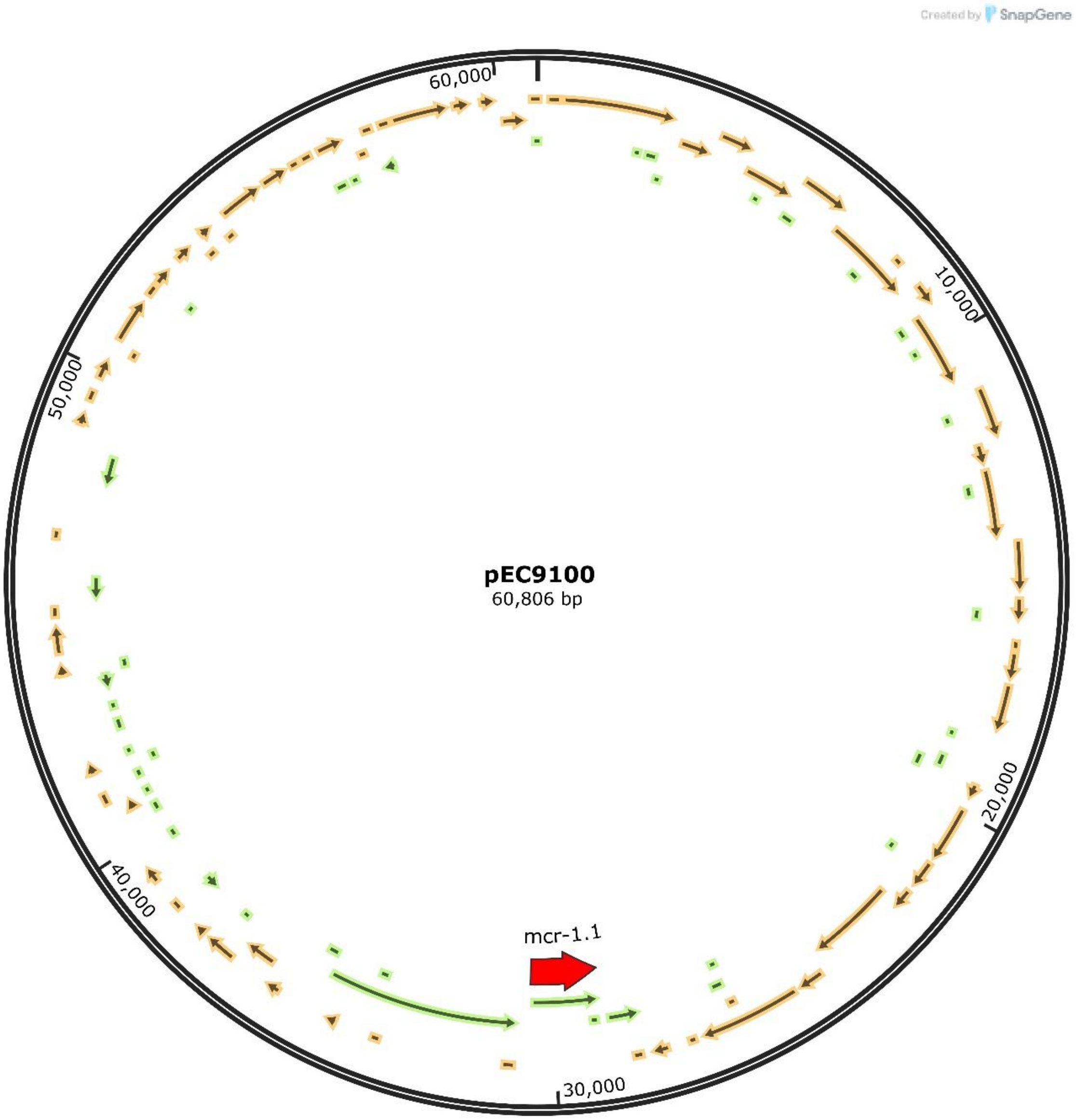

Supplementary Figure 3. Plasmid pEC9100 map showing the annotations for regions containing *mcr-1.1*. Detailed segment annotation is depicted in Figure 1(C and E) and table 1.

Plasmid “pEC9116B” recovered from the strain: **SAMN48080415** harboring *mcr-1.1*

Typing: IncI2

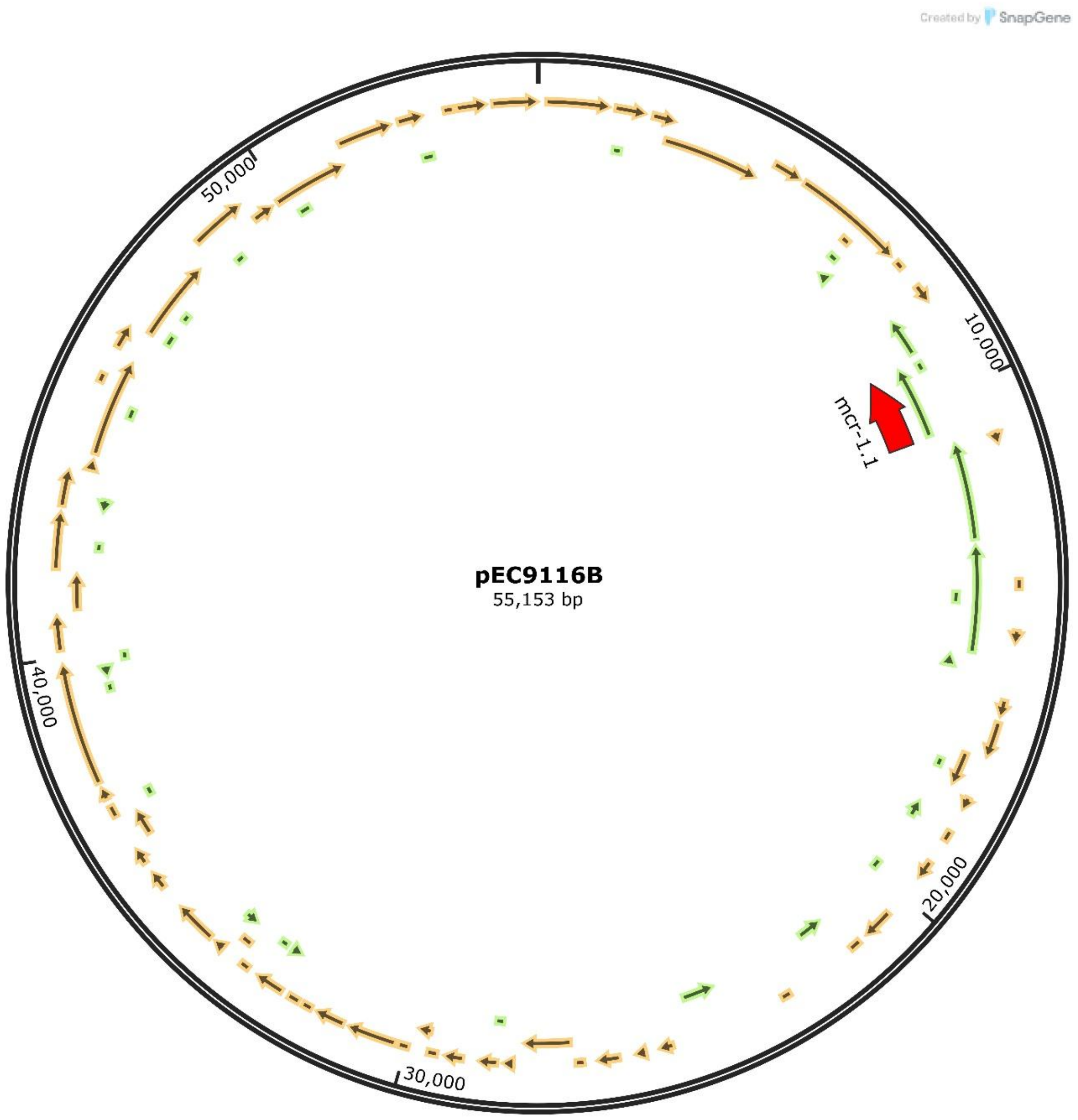

Supplementary Figure 4. Plasmid pEC9116B map showing the annotations for regions containing *mcr-1.1*. Detailed segment annotation is depicted in Figure 1(C and E) and table 1.

Plasmid “pEC9116A” recovered from the strain: **SAMN48080415** harboring *tet(X4)*

Typing: IncF Megaplasmid [IncFIB(AP001918)-IncFII]

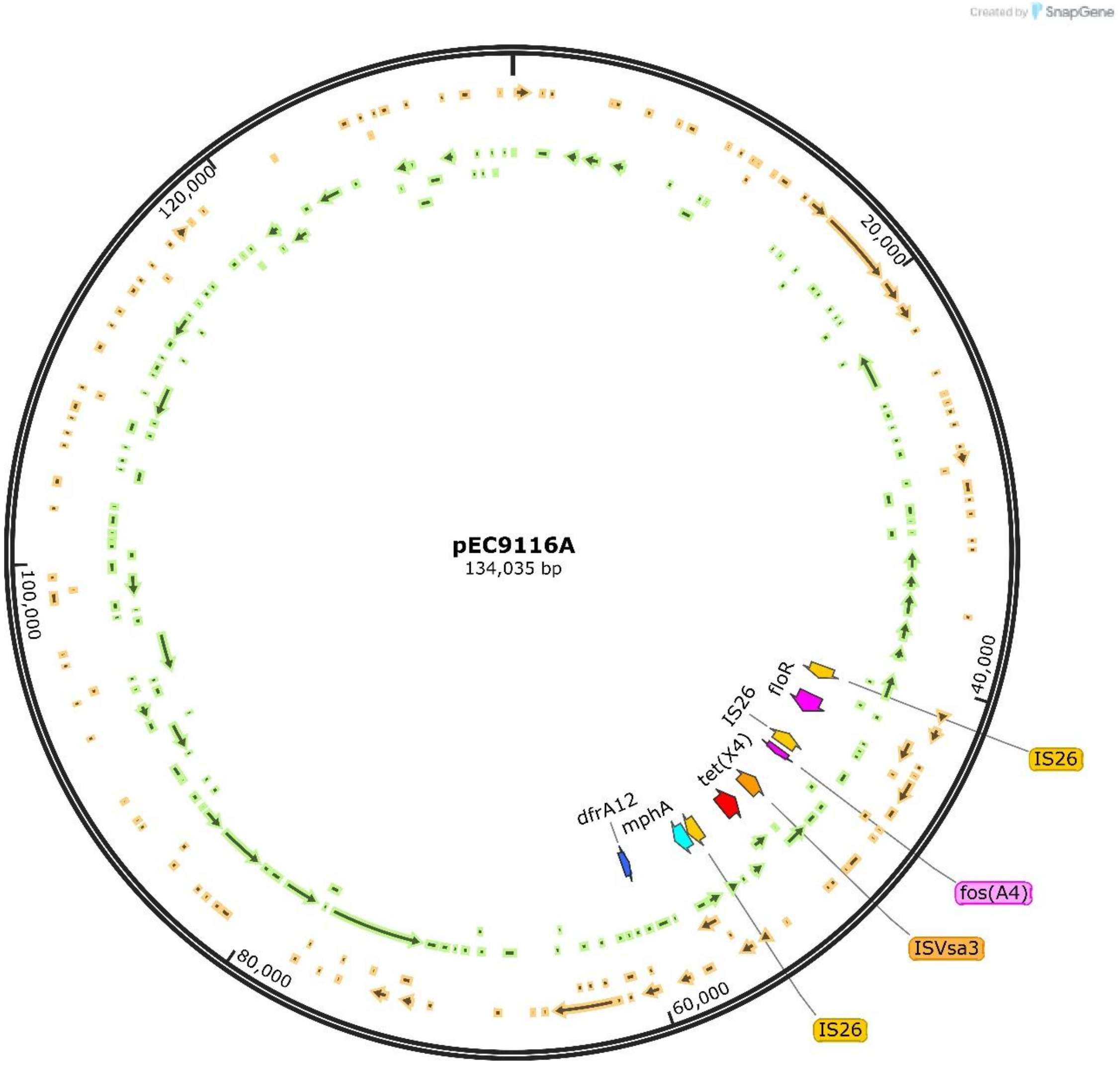

Supplementary Figure 5. Plasmid pEC9116A map showing the annotations for regions containing *tet(X4)* and *fos(A4)* with all related IS regions. Detailed segment annotation is depicted in Figure 1(B and D) and table 1.
